# An Optimized Method for Batch Recovery Based on Erasure Coding in Heterogeneous Network

**DOI:** 10.3390/s25020346

**Published:** 2025-01-09

**Authors:** Ying Song, Jialin Liu, Yingai Tian, Bo Wang

**Affiliations:** 1 School of Computer Science, Beijing Information Science and Technology University, Beijing 102206, China; 2023020647@bistu.edu.cn (J.L.); tianyingai@bistu.edu.cn (Y.T.); 2 Software Engineering College, Zhengzhou University of Light Industry, Zhengzhou 450002, China; wangb@zzuli.edu.cn

**Keywords:** bandwidth management, load balancing, distributed storage system, heterogeneous network, erasure coding, batch recovery, Internet of Things

## Abstract

With the rapid development of IoT technology, sensors are widely used for monitoring environmental parameters. The data collected by sensors needs to be stored, and distributed storage systems provide an excellent platform to handle this vast amount of data. To enhance data reliability and reduce storage costs, erasure coding technology can be employed within distributed storage systems. However, the process of recovering lost or damaged data inevitably generates significant cross-rack traffic. In recent years, various batch recovery methods have been designed to improve data recovery speed and reduce cross-rack recovery traffic, but they have limitations in different aspects, such as less consideration for heterogeneous cross-rack bandwidth and insufficient handling of recovery link scheduling. This paper proposes the HBRepair recovery framework, aimed at improving data recovery speed by balancing the recovery load and reducing cross-rack recovery traffic. Firstly, HBRepair strategically selects helper blocks and nodes for storing recovery blocks to find the optimal batch recovery plan. Then, it selectively and rationally schedules recovery links to saturate idle bandwidth resources and avoid network congestion. Experimental results show that by optimizing the use of cross-rack bandwidth, HBRepair can reduce cross-rack recovery time by up to 26.74%, effectively addressing the shortcomings of existing methods.

## 1. Introduction

With the widespread adoption of the internet and the rapid development of the Internet of Things (IoT), the demand for the collection, storage, and processing of large amounts of data is increasingly growing. In sensor networks, sensors serve as core components, responsible for collecting data and transmitting them to the central node of the network to enable the monitoring and management of the physical environment. However, the large number of sensors in the network, each generating a large amount of data, has led to a sharp increase in data volume, making the efficient storage and management of this vast amount of data an urgent issue to be addressed. If data are simply stored at the central node, they may not only cause excessive consumption of storage resources, but also decrease the network’s transmission speed and response speed. Therefore, to efficiently manage the data produced by sensor networks, adopting a distributed storage system to handle these substantial data volume is a more professional and reasonable choice. This strategy has also been implemented in practical applications, such as Amazon Web Services (AWS) IoT, Microsoft Azure IoT Hub, and Google Cloud IoT, which are all examples of using distributed storage systems to optimize the management of data within sensor networks.

In distributed storage systems, erasure coding can provide the same level of data reliability at a lower storage cost compared to multiple replicas. Due to its efficient storage efficiency, erasure coding is widely applied in distributed storage systems, such as Facebook’s HDFS storage system [1] and Windows’ Azure Storage [2,3]. Among various erasure codes, the Reed-Solomon (RS) code is the most commonly used. When applying RS codes, a file is divided into *k* original blocks (referred to as data blocks), and *m* redundant blocks (referred to as parity blocks) are generated through encoding. These *k + m* blocks are stored across *k + m* nodes, forming a stripe. Data centers typically store multiple stripes, and each stripe is independently encoded based on the specific construction of the erasure code. However, a major drawback of erasure coding is the high cost of data recovery. In the event of a loss or damage to a data block or parity block, erasure coding requires retrieving *k* surviving blocks as helper blocks to recover the damaged block.

Addressing this shortcoming, existing research has developed a series of related recovery techniques, such as PPR [4], RepairBoost [5], CMRepair [6], ClusterSR [7], SelectiveEC [8], HMBR [9], BPR [10], the Multi-Block Double-Tree Update (MDTUpdate) technique [11] and SDNC-Repair [12], etc., to improve the data recovery performance of erasure codes. However, they have limitations in the following three aspects.

Firstly, existing studies overlook the rack architecture and heterogeneous networks, randomly selecting helper blocks and target nodes without considering the generation of a near-optimal multi-strip recovery scheme. In practical applications, nodes in an erasure coding cluster are typically distributed across multiple racks, with nodes within each rack connected through an internal switch, and all racks connected through a core switch. In addition, there are differences in the link bandwidth of the erasure code cluster [13]. Within a rack, bandwidth is generally considered to be sufficient, whereas cross-rack bandwidth is often scarce [14]. Neglecting the rack architecture may result in large-scale cross-rack data transfers during data recovery, thereby reducing overall recovery performance. Ignoring the performance differences between racks, treating the cross-rack network as homogeneous, can result in the inability to allocate network resources evenly in actual heterogeneous network environments, thus decreasing the efficiency of data transfer and recovery.

Secondly, existing research has not effectively scheduled recovery links to fully utilize bandwidth. In heterogeneous networks, recovery links with different transmitter racks and receiver racks have varying transmission times. Analysis reveals that randomly scheduling recovery links may lead to network congestion, which affects recovery performance.

Finally, existing recovery technologies lack flexibility and cannot be directly deployed to different erasure codes or recovery algorithms to adapt to various recovery environments.

This paper addresses these issues by designing HBRepair, a recovery framework that aids in accelerating batch recovery for various erasure codes and recovery algorithms.

The main idea of HBRepair is to first formalize the recovery scheme for a single block by creating a directed acyclic graph (HBDAG) that represents the specified data transfer directions in the recovery algorithm. Then, HBRepair balances the recovery traffic and bandwidth utilization in batch recovery through the following two designs. (i) It allocates the recovery tasks of multiple blocks to the nodes of upload helper blocks and target nodes based on the HBDAG, to balance the overall upload and download recovery traffic. (ii) It schedules the execution order of recovery links reasonably to saturate the upload and download bandwidth. In summary, the main contributions of this paper are as follows.

- [Design] This paper proposes the HBRepair recovery framework to assist existing erasure codes and recovery algorithms in order to accelerate batch recovery. HBRepair formalizes single-block recovery tasks through HBDAGs, and then balances the upload and download recovery traffic by assigning recovery tasks to specific nodes. HBRepair also optimizes the execution order of recovery links to saturate unoccupied bandwidth (Section 3.2, Section 3.3 and Section 3.4).
- [Implementation] The prototype of HBRepair is implemented in C++, and it can be deployed as an independent middleware on existing storage systems for recovery scheduling.
- [Evaluation] This paper evaluates the performance of HBRepair on Alibaba Cloud, demonstrating that it can support a variety of erasure codes and recovery algorithms. Compared to RepairBoost [5] and CMRepair [6], HBRepair can reduce the recovery time by 11.64–26.74% (Section 5).

The rest of this paper is as follows. Section 2 introduces the research background of this paper and presents the limitations of existing related work, which motivates the design of HBRepair. Section 3 describes the design of HBRepair in detail. Section 4 discusses the generality and flexibility of HBRepair in integrating with different erasure codes and recovery algorithms. Section 5 presents the evaluation results and performance analysis of HBRepair. Finally, Section 6 summarizes the main contributions of this paper and outlines directions for future work.

## 2. Related Work

The related work section begins with a brief review of the fundamental properties of erasure coding (Section 2.1). Existing batch recovery methods either simply repeat the single-stripe recovery approach, leading to high upload (download) recovery traffic, unbalanced load, reduced stripe recovery efficiency, and wastage of additional resources. Or they overlook the heterogeneous cross-rack bandwidth, lack special handling for the scheduling of recovery links, and are constructed only for specific erasure codes when designing optimization strategies for global recovery tasks. Therefore, this paper focuses on batch recovery optimization methods for global recovery tasks and provides a detailed introduction to the current state of erasure codes in batch recovery methods (Section 2.2). Finally, this paper also summarizes the limitations of existing studies (Section 2.3).

In order to help readers better understand the professional terms in the following text, this paper provides a detailed explanation of these terms through Table 1 before starting Section 2.

### 2.1. Erasure Coding

Erasure coding reduces storage overhead while ensuring reliability by introducing a small amount of computational operations. Erasure coding is configured with two integer parameters (namely *k* and *m*) to balance storage overhead and fault tolerance. Erasure coding involves two operations: encoding and decoding. In the encoding phase, erasure coding encodes *k* data blocks to generate additional *m* check blocks. These *k + m* blocks form a stripe, where any *k* blocks (data blocks or parity blocks) within the stripe can be used to recover the damaged block. This means that erasure coding can tolerate any *m* block failures within each stripe. Therefore, by distributing the *k + m* blocks of each stripe across *k + m* nodes, with each node storing one block, erasure coding can tolerate the failure of any *m* nodes. Moreover, this paper ensures rack-level fault tolerance by limiting each rack to store at most *m* blocks of a single stripe [15,16]. This is performed because it is always possible to obtain at least *k* surviving blocks of the same stripe from other available racks.

In this paper, for ease of understanding, the Reed-Solomon code (RS code) is primarily used as an example because of its widespread application in practice [17,18,19,20,21]. However, the methods presented in this paper can be readily extended to other erasure codes (Section 5.2), such as Locally Repairable Codes (LRC) and Minimum Storage Regenerating Codes (MSR).

### 2.2. Batch Recovery Methods Based on Erasure Coding

Batch recovery requires recovery across multiple stripes. Although erasure codes have high storage efficiency, they can incur high recovery costs. For example, RS(*k*,*m*) requires the transmission of *k* surviving blocks within the same stripe to recover a damaged block, thus the storage I/O and network I/O will be magnified by a factor of *k* during the recovery process. Specifically, assume block *C′* fails, and $\left\{C_{1},C_{2},\ldots ,C_{k}\right\}$ are the *k* blocks in the same stripe, then these *k* blocks can be used to recover linearly as shown below.

(1) $$C^{\prime }=\sum_{i=1}^{k}\beta_{i}C_{i}$$

where $\beta_{i}$ represents the decoding coefficients used by $C_{i}$ to recover *C′*. In light of the high recovery cost, existing research has developed the following schemes to reduce the recovery cost. These batch recovery schemes can be roughly categorized into two types: one type achieves batch recovery by repeating the recovery method of a single stripe, and the other type is an optimization strategy for batch recovery that targets global recovery tasks.

**Figure 1:**
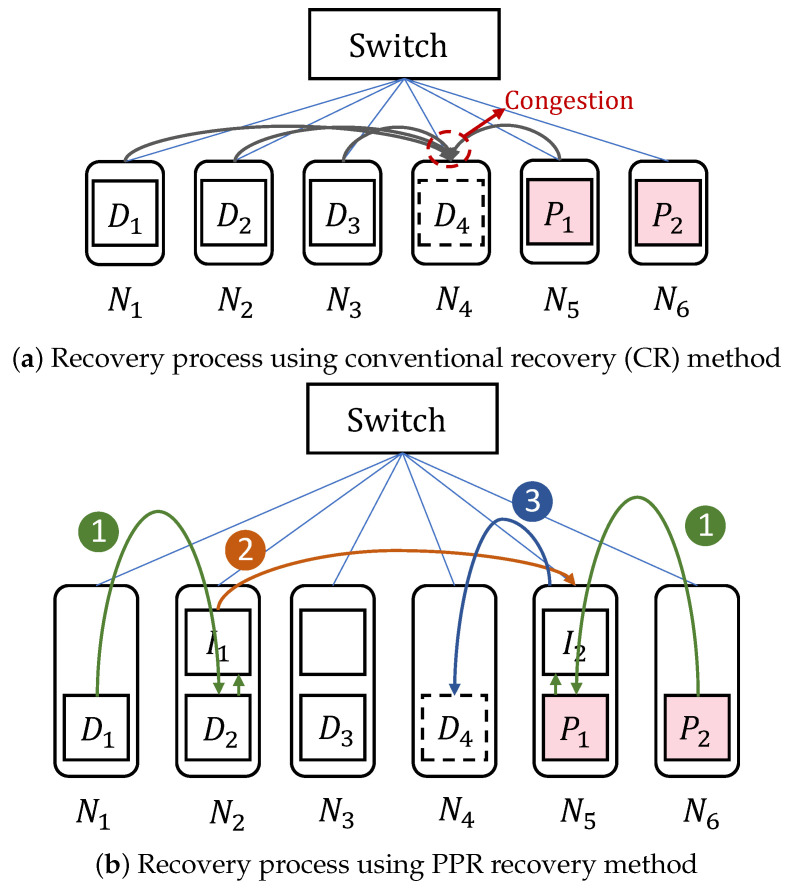
Process of recovering damaged block using different recovery methods.

**The recovery method that repeats a single stripe:** Existing single-stripe recovery algorithms primarily focus on how to quickly transmit surviving blocks, how to obtain the optimal recovery scheme, and how to schedule recovery links. Recovery algorithms that focus on the rapid transmission of surviving blocks utilize idle bandwidth to accelerate recovery without reducing the recovery traffic. Figure 1a shows the recovery process for damaged block $D_{4}$ using the conventional recovery method (CR) with RS(4,2) coding. When using the CR method for single-stripe recovery, all helper blocks are transmitted to the target node simultaneously, without involving any specific design optimizations or strategies. As shown in Figure 1a, this process requires transmitting 4 surviving blocks $\left\{D_{1},D_{2},D_{3},P_{1}\right\}$ to the target node (the node where the recovered block is stored) $N_{4}$. Obviously, using conventional recovery can cause congestion in the download link of the node, so it takes four time slots to recover the damaged block. PPR [4] decomposes single-block recovery into multiple sub-phases and executes these sub-phases in parallel to alleviate network congestion, thereby achieving the goal of fully utilizing available bandwidth. Figure 1b describes the recovery process for the damaged block $D_{4}$ using PPR [4] recovery with RS(4,2) coding. It can complete the recovery in the following three time slots. (i) In the first time slot, $D_{1}$ is sent to $D_{2}$, and it is linearly combined with $D_{2}$, as shown in Equation (1), to generate the intermediate block $I_{1}$, while $P_{2}$ is sent to $P_{1}$ to generate another intermediate block $I_{2}$. (ii) In the second time slot, $I_{1}$ is sent to $I_{2}$, and $I_{1}$ is linearly combined with $I_{2}$ to recover the damaged block $D_{4}$. (iii) In the third time slot, the recovered block $D_{4}$ is sent to the storage node $N_{4}$. Therefore, PPR [4] can fully utilize the available bandwidth in each time slot to mitigate network congestion issues in single-block recovery. However, PPR [4] overlooks the rack architecture, which may trigger extensive cross-rack data transfers and impact recovery performance. ECPipe [22] further reduces the recovery time to a single time slot by breaking down the recovery of the damaged block into multiple smaller sub-blocks for pipelined recovery. When these two methods are applied to batch recovery, they simply repeat the recovery method of a single stripe. This approach fails to fully utilize the bandwidth in each time slot, which may inadvertently cause link congestion and reduce bandwidth utilization.

**Optimization strategies for global recovery tasks:** Existing optimization strategies for global recovery tasks include RepairBoost [5], CMRepair [6], ClusterSR [7], SelectiveEC [8], HMBR [9], and SDNC-Repair [12]. RepairBoost [5] is a recovery framework that balances upload and download recovery traffic by carefully assigning recovery tasks to each node. In addition, it utilizes the maximum flow problem to arrange the execution order of recovery links to fully utilize the idle bandwidth. However, the strategy focuses on node-level recovery within racks, ignoring the rack architecture. This may lead to the overutilization of the scarce bandwidth, subsequently causing network congestion and reducing the speed of recovery. CMRepair [6] reduces recovery time by initializing and optimizing the recovery scheme and selectively scheduling recovery links to saturate upload/download bandwidth resources, thereby avoiding network congestion. However, this method cannot flexibly combine different recovery algorithms to meet the reliability requirements of various storage systems in practice. ClusterSR [7] first determines the recovery scheme for each stripe based on the data distribution and then iteratively adjusts the recovery scheme for multiple stripes using greedy algorithms to arrange the recovery of multiple stripes. However, when adjusting the recovery scheme for multiple stripes, the method may necessitate reconfiguring the layout of data blocks, resulting in a decrease in recovery efficiency. SelectiveEC [8] optimizes the recovery process by generating the optimal recovery scheme on a bipartite graph. However, the method does not consider the reasonable arrangement of the execution order of recovery links, which may lead to multiple upload (download) links multiplexing the sender’s (receiver’s) bandwidth, causing network congestion and affecting recovery performance. HMBR [9] is a hybrid multi-block recovery mechanism that combines both centralized and decentralized recovery methods to balance the bandwidth bottleneck of upload/download data nodes, thereby improving the efficiency of multi-block recovery. Yet, this method cannot be applied in heterogeneous network environments, limiting its performance and efficiency in practical applications. SDNC-Repair [12] designs data source selection algorithms and data flow scheduling algorithms to shorten data transmission time and enhance system reliability. However, when generating recovery schemes, this method only considers the selection of data sources, which means it only takes into account the load of the upload nodes and neglects the load of the download nodes. This may lead to an excessive recovery traffic load on a certain download node, making it a bottleneck in the recovery process, which in turn leads to unbalanced link loads, network congestion, and impacts the recovery time.

### 2.3. Discussion on the Limitations of Related Work

By analyzing the existing methods for accelerating erasure code recovery, this paper identifies the following limitations, which, if not properly addressed, will restrict their performance in batch recovery applications.

**Figure 2:**
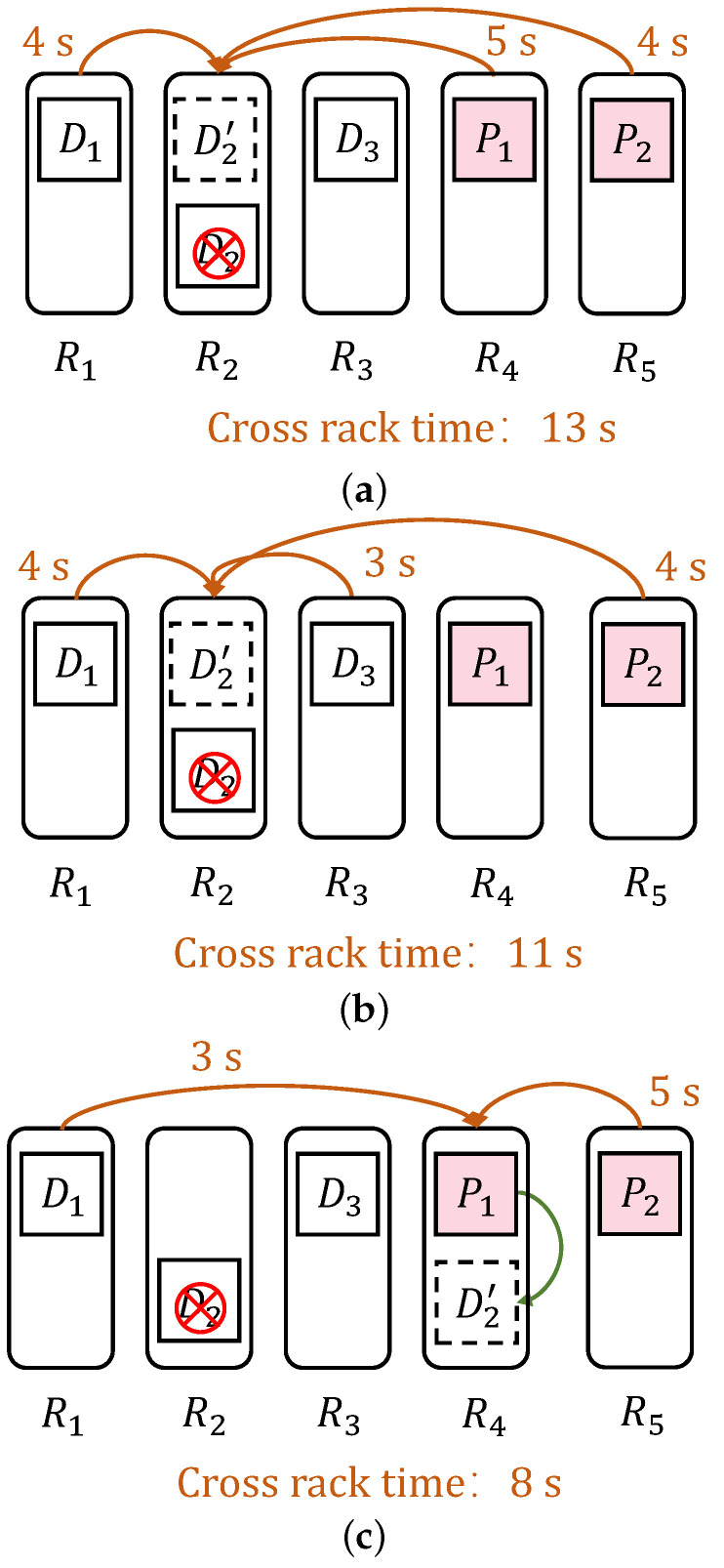
Cross-rack transmission time for different recovery schemes using the CR method. (**a**) Using the CR method to randomly select help blocks and target nodes for recovery scheme; (**b**) Using the CR method to select a recovery scheme different from the help blocks in (**a**); (**c**) Using the CR method to select a recovery scheme different from the target node in (**a**).

**Limitation** **1.** *No approach generates a near-optimal multi-strip recovery scheme. The recovery scheme specifies the helper blocks used for recovery and the nodes for storing the recovered blocks, which determines the time for cross-rack data transfer. The following is an illustration with an example. There is currently a stripe encoded with RS(3,2) that consists of 5 blocks $\left\{D_{1},D_{2},D_{3},P_{1},P_{2}\right\}$, assuming $D_{2}$ is corrupted. Table 2 shows the cross-rack transfer times, and Figure 2a–c depict three different recovery schemes. Specifically, the random recovery scheme in Figure 2a selects $\left\{D_{1},P_{1},P_{2}\right\}$ as helper blocks and stores the recovered block in rack $R_{2}$. Due to the scarcity of cross-rack bandwidth and network contention, multiple download (upload) links cannot simultaneously transfer data blocks. Therefore, the random recovery scheme requires 13 s for recovery. In comparison, Figure 2b shows a recovery scheme with $\left\{D_{1},D_{3},P_{2}\right\}$ as helper blocks, which requires 11 s, and Figure 2c shows a recovery scheme with $R_{4}$ as the storage rack, which requires a recovery time of 8 s. It is important to note that blocks from the same stripe cannot be located on the same node, and a rack can store at most m blocks from the same stripe. This example demonstrates that selecting a near-optimal recovery scheme can reduce cross-rack transfer time, thereby reducing the recovery time.*

**Figure 3:**
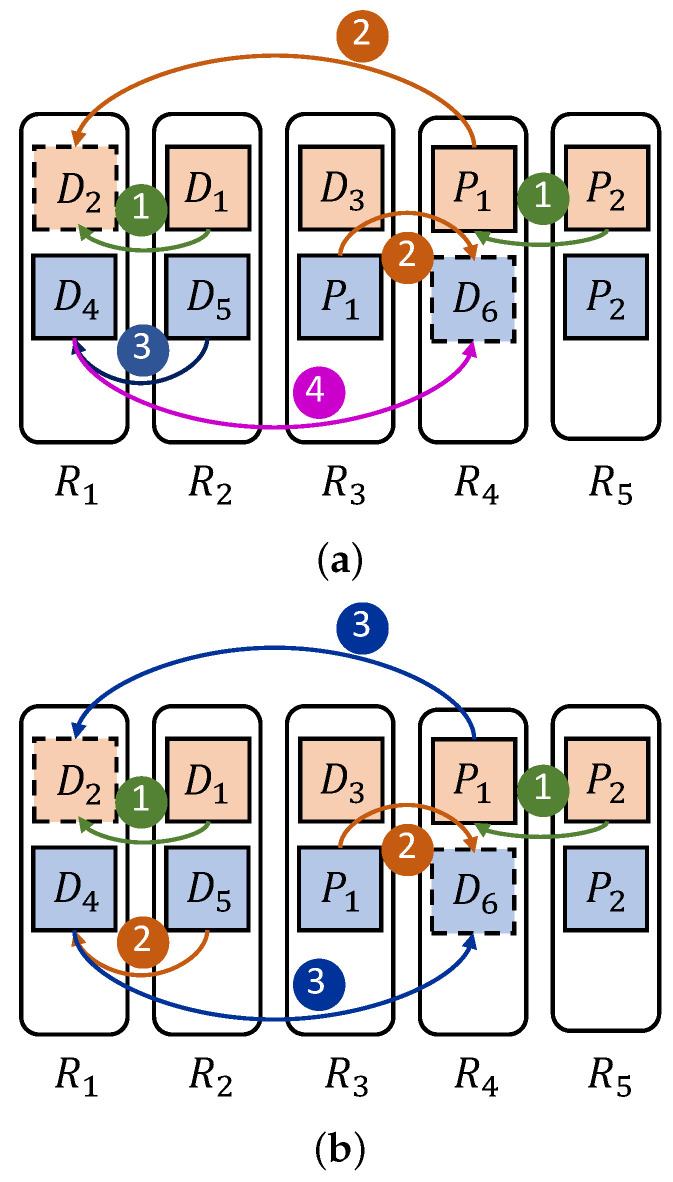
The impact of scheduling sequence for restoring links on bandwidth utilization; (**a**) Complete the recovery process in 4 time slots; (**b**) Complete the recovery process in 3 time slots.

**Limitation** **2.** *Inadequate utilization of bandwidth for each time slot. Although existing recovery algorithms can alleviate download bottlenecks in single-block recovery, e.g., PPR [4] and ECPipe [22], they simply repeat the recovery method of a single stripe to handle batch recovery. This may inadvertently lead to link congestion and reduced bandwidth utilization during recovery. Figure 3 shows the impact of scheduling recovery links reasonably on the recovery process. As shown in Figure 3a, after transmitting $D_{1}$ and $P_{2}$ in the first time slot, the transmission of $P_{1}$ (from rack $R_{4}$ to $R_{1}$) and $D_{5}$ (from rack $R_{2}$ to $R_{1}$) will compete for the bandwidth of $R_{1}$’s download link. Figure 3a selects the priority transmission of $P_{1}$ in the second time slot. Since $D_{4}$ can only be sent after $D_{5}$ is received, Figure 3a ultimately requires 4 time slots to transfer the data blocks. In contrast, Figure 3b selects to send $D_{5}$ in the second time slot, allowing $P_{1}$ and $D_{4}$ to be transmitted in parallel without bandwidth competition. Therefore, Figure 3b only needs 3 time slots to transfer the data blocks. This example indicates that the execution order of recovery links should be arranged reasonably during batch recovery.*

**Limitation** **3.** *Lack of a general framework for batch recovery. Existing distributed storage systems deploy different specific recovery algorithms for single-block recovery. For example, many commercial storage systems still rely solely on CR for single-block recovery without considering the use of PPR [4] in tiered storage systems to reduce cross-rack recovery traffic. Therefore, designing a general recovery framework that supports the application of different types of recovery algorithms in various deployment scenarios is crucial.*

## 3. Design of HBRepair

This paper proposes a recovery framework named HBRepair, aimed at balancing the recovery load, reducing cross-rack recovery traffic, and improving recovery speed.

**Assumption** **1.** *HBRepair is designed based on the following assumptions. Firstly, HBRepair primarily focuses on the single-block recovery within each stripe, as this is the most prevalent fault in practice (accounting for approximately 98% of all failures) [23]. Secondly, to simplify the discussion, this paper elaborates on HBRepair’s application to common recovery methods (CRs) using RS codes in heterogeneous environments, but HBRepair is also applicable to other erasure codes and can be deployed in other recovery algorithms (Section 5.2).*

HBRepair employs the following techniques to address the aforementioned issues (Section 2.3). Firstly, HBRepair selects nearly optimal recovery schemes for each stripe, which involves choosing the best nodes for uploading helper blocks and target nodes for each stripe, to reduce cross-rack transmission time and total recovery time.

Then, HBRepair abstracts single-block recovery schemes through directed acyclic graphs (HBDAG) and achieves recovery for multiple stripes by scheduling multiple HBRDAG. HBRepair’s flexibility and generality, i.e., applicability to various erasure codes and recovery algorithms, are based on this approach.

Finally, HBRepair finds a reasonable scheduling order for all recovery links to saturate idle upload and download bandwidth resources, thus avoiding network congestion.

Next, to clarify the research objectives and simplify complex issues into a more analytically and understandable theoretical model, this paper first provides a formal description of the problems HBRepair aims to solve. Subsequently, this paper introduces two design techniques of HBRepair in detail: one is how to generate nearly optimal recovery schemes, and the other is how to schedule recovery links reasonably.

### 3.1. Formalization of Problems

Assume a cluster encoded with RS(*k*,*m*) is deployed across *n* nodes, denoted as $\left\{N_{1},N_{2},\ldots ,N_{n}\right\}$. These nodes are evenly distributed across *r* racks, represented as $\{R_{1},R_{2},$$\ldots ,R_{r}\}$. The cluster contains *s* stripes with corrupted blocks, with each data block of stripe $S_{i}$ distributed across *k + m* different nodes. Let $b_{i,j}$ represent the data block of stripe $S_{i}$ stored on node $N_{j}$, and $solu_{i}$ represent the recovery scheme for stripe $S_{i}$. The $solu_{i}$ consists of *k* + 1 elements, with the first *k* elements indicating helper blocks, and the last element indicating the rack where the recovery block is stored. It is important to note that to ensure rack-level fault tolerance, the recovery block must be stored on a node that does not contain other data blocks of the same stripe. This paper also sets a cost value $Cost_{mn}$ for each cross-rack link $R_{m}$ → $R_{n}$, which is the time taken to transmit a data block over the cross-rack link $R_{m}$ → $R_{n}$. In this paper, the cost values are set in the experimental environment configuration. Let $Tu_{i}$ and $Td_{i}$ be the upload and download times of rack $R_{i}$, respectively, then the maximum upload/download time $T_{i}$ of rack $R_{i}$ can be expressed as $T_{i}$ = max($Tu_{i}$, $Td_{i}$). Therefore, the maximum transmission time *MT* across all racks can be expressed as follows.

(2) $$MT=\max(T_{1},T_{2},\ldots ,T_{r})$$

Through further observation of Figure 3, it can be known that after arranging the execution order of recovery links reasonably, the minimum recovery time for batch recovery can be equal to the maximum transmission time across all racks. If recovery links are scheduled randomly, the minimum recovery time for batch recovery may exceed the maximum transmission time across all racks. Therefore, the relationship between the minimum recovery time for batch recovery *BT* and the maximum transmission time *MT* can be expressed as follows.

(3) BT≥MT

Due to the sparsity of cross-rack bandwidth and the fact that cross-rack transmission time accounts for a significant portion of the total recovery time (about 85–90%), this paper primarily aims to reduce the recovery time *BT* by decreasing the maximum transmission time *MT* across all racks. Hence, the optimization objective can be formulated as follows.

(4) MinimizeMT→MinimizeBT

### 3.2. Generate Recovery Scheme

In the batch recovery problem, if the optimal recovery scheme is selected from all possible schemes using an enumerative approach, it will result in an algorithm with excessively high time complexity. Therefore, this paper employs a greedy algorithm to iteratively replace the current recovery scheme with another one that has a shorter maximum transmission time *MT*, in order to find a near-optimal recovery scheme.

**Algorithm details:** Algorithm 1 presents the pseudocode, which includes two phases: initializing the recovery scheme and optimizing the recovery scheme. Before elaborating on the specific steps of Algorithm 1, it is necessary to explain that the number of lines in the following description refers to the number of lines of code in Algorithm 1.
**Algorithm 1** Generate repair solution**Input:** number of stripes *s*, number of racks *r***Output:** a multi-stripe repair solution *Solu*
       //Initialize repair solution
 1:   **for** i=1 to *s* **do** 2:      Select an optimal single-stripe solution $solu_{i}$ for stripe $S_{i}$ 3:   Initialize a multi-stripe solution *Solu* = $\left\{solu_{1},\ldots ,solu_{s}\right\}$    // Solution optimization 4:   **while** each optimization **do** 5:      Compute *MT* of *Solu* 6:      Locate rack $R_{x}$ with $T_{x}$ = *MT* 7:      **for** each $solu_{i}$ in *Solu* **do** 8:         **if** $Tu_{x}$ = *MT* **then** 9:            **if** $solu_{i}$ retrieves helper blocks from $R_{x}$ **then**10:               **for** j=1 to *r* **do**11:                  **if** $R_{j}\neq R_{x}$ and $R_{j}$ contains other helper blocks **then**12:                     Generate a new single-stripe solution $solu_{i}$′ for stripe $S_{i}$13:         **else if** $Td_{x}$ = *MT* **then**14:            **if** $solu_{i}$ chooses $R_{x}$ as the requestor **then**15:               **for** j=1 to *r* **do**16:                  **if** $R_{j}\neq R_{x}$ **and** $R_{j}$ can be the requester **then**17:                     Generate a new single-stripe solution $solu_{i}$′ for stripe $S_{i}$18:         nSolu = Substitutin(*Solu*, $solu_{i}$′, i)19:         Compute *nMT* of *nSolu*20:         **if** *nMT* < *MT* **then**21:            *Solu* = *nSolu*22:            Jump to the next optimization of the while-loop in line 423:         **else if** $R_{j}$ can find other $solu_{i}$′ **then**24:            Jump to the line 12 ($Tu_{x}$ = *MT*) or line 17 ($Td_{x}$ = *MT*)25:         **else**26:            Jump to the line 10 ($Tu_{x}$ = *MT*) or line 15 ($Td_{x}$ = *MT*)            until cannot find the $solu_{i}$′ for stripe $S_{i}$27:      Exit the while-loop in line 4 if there is no substitution in *Solu*28:   **return** Solu

**Initialize the recovery scheme.** First, find the best single-stripe recovery scheme $solu_{i}$ for the i-th stripe $S_{i}$ (Lines 1–2 in Algorithm 1). Currently, there are many methods applied in heterogeneous environment to reduce the single-stripe recovery time [13,24]. However, only AZ-recovery [24] considers the rack architecture, so it is chosen as the basis for the initialization of the recovery scheme in this paper. For a single stripe, AZ-recovery identifies the optimal recovery scheme with the shortest Maximum Transmission Time *MT* by enumerating all valid recovery schemes. Since each stripe has at most C(k+m−1,k)·r valid recovery schemes, Algorithm 1 needs to enumerate at most C(k+m−1,k)·r·s recovery schemes to obtain a near-optimal recovery scheme during batch recovery. In contrast, the enumerative approach would require enumerating ${\left(C\left(k+m-1,k\right)\cdot r\right)}^{s}$ recovery schemes to find the best recovery scheme. Specifically, C(k+m−1,k) is the number of optional helper blocks, *r* is the number of racks that can store recovery blocks, and *s* is the number of stripes. This demonstrates that Algorithm 1 can significantly reduce the time for initializing the recovery scheme during batch recovery. Then, initialize a multi-stripe recovery scheme *Solu* (Line 3), which consists of *s* optimal single-stripe recovery schemes.

**Optimize the recovery scheme.** After constructing the initial multi-strip recovery scheme *Solu*, a greedy algorithm is applied to iteratively optimize *Solu* to reduce the Maximum Transfer Time *MT* (Lines 4–27). Specifically, in each optimization, first calculate *MT* based on *Solu* and locate the rack $R_{x}$ with the maximum upload or download time, which is the rack where $T_{x}$ = *MT* (Lines 5–6). Next, iterate through each single-strip recovery scheme $solu_{i}\in Solu$ (Line 7). If the upload time of rack $R_{x}$ equals *MT* ($Tu_{x}$ = *MT*), and the current single-strip recovery scheme $solu_{i}$ requires obtaining a helper block from rack $R_{x}$, then it is necessary to find another valid recovery scheme $solu_{i}$′ in other racks (Lines 8–12). By replacing $solu_{i}$ with $solu_{i}$′ in *Solu*, a new multi-strip recovery scheme *nSolu* is generated (Line 18). Recalculate the maximum transfer time *nMT* of *nSolu* (Line 19). If *nMT* < *MT*, replace *Solu* with *nSolu* and jump back to the while loop at line 4 for the next optimization (Lines 20–22). If nMT≥MT, and there are other valid recovery schemes in the currently iterated rack, jump to line 12 to find another valid recovery scheme $solu_{i}$′ that meets the conditions (Lines 23–24). If nMT≥MT, and there are no other valid recovery schemes in the currently iterated rack $R_{j}$, jump to line 10 to find another valid recovery scheme $solu_{i}$′ in other racks (Lines 25–26). The reduction in download time for rack $R_{x}$ is also similar (Lines 13–17). After each iteration, the Maximum Transfer Time *MT* will decrease monotonically. Algorithm 1 will repeat the aforementioned replacements until *Solu* is no longer replaceable (Line 27). Ultimately, Algorithm 1 will return the optimal multi-strip recovery scheme *Solu* (Line 28).

**Figure 4:**
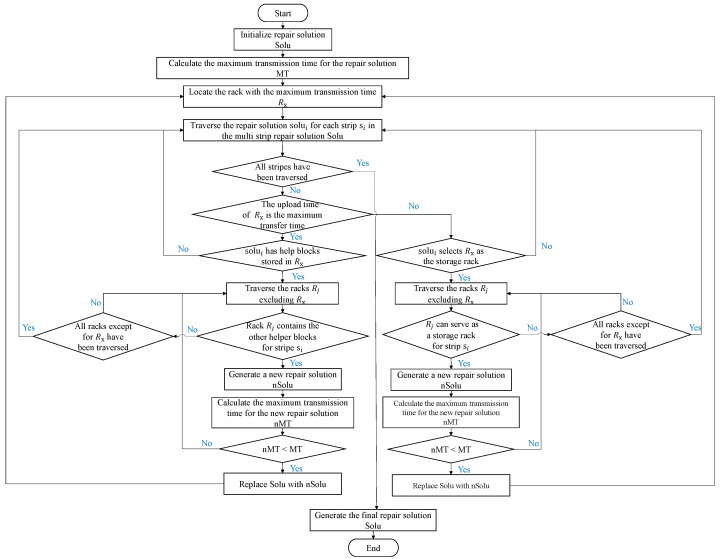
Flowchart of Algorithm 1.

**Algorithm flowchart:** In order to help readers better understand the process of Algorithm 1, this paper provides a flowchart of the algorithm, as shown in Figure 4.

**Figure 5:**
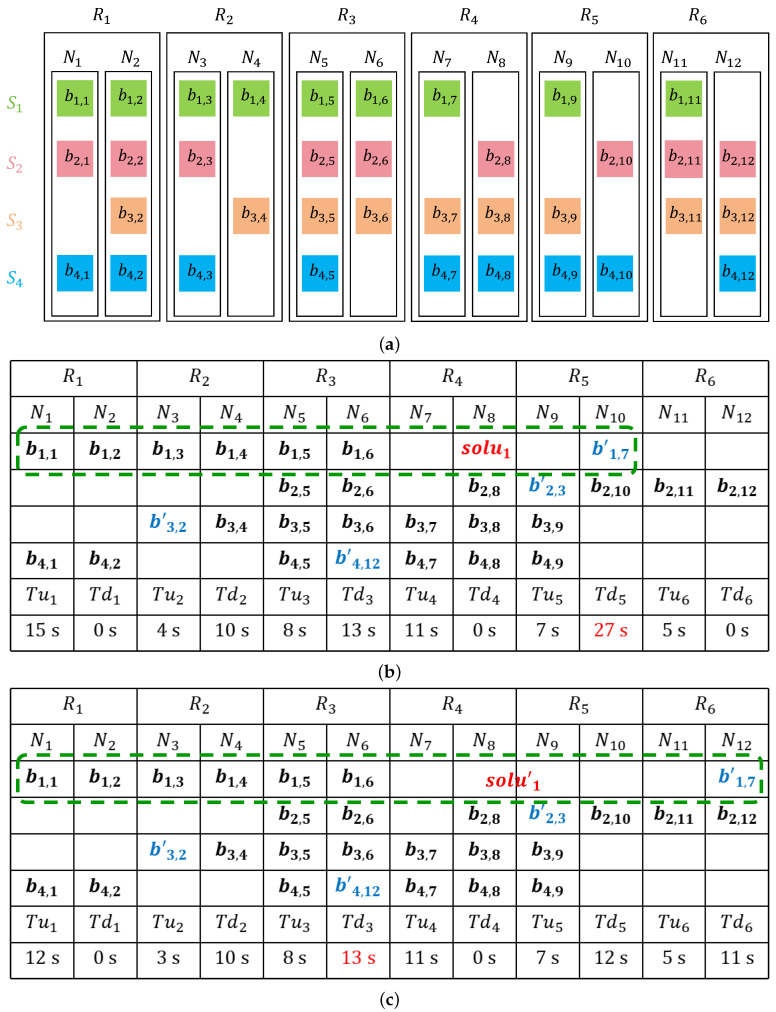
Example of Algorithm 1 for generating repair solution. (**a**) Data block layout in the example; (**b**) Recovery scheme without optimization; (**c**) Optimized recovery scheme.

**Example analysis:** To facilitate understanding, this paper elucidates Algorithm 1 through an example. As shown in Figure 5a, there are 4 failed strips: $S_{1}$, $S_{2}$, $S_{3}$, and $S_{4}$. The cluster, which employs RS(6,3) encoding, consists of 6 racks and 12 nodes. Figure 5a and Table 3 display the data block layout and the transmission times between racks, respectively. Assume data blocks $b_{1,7}$, $b_{2,3}$, $b_{3,2}$, and $b_{4,12}$ are damaged. From Figure 5b, it can be observed that rack $R_{5}$ has the maximum download time ($Tu_{5}$ = *MT* = 27 s), and there are two strip recovery schemes that select rack $R_{5}$ to store the recovery blocks. Therefore, the recovery process is constrained by the downlink of rack $R_{5}$. To reduce the download time of $R_{5}$, rack $R_{6}$ is chosen as the rack to store the recovery blocks for $solu_{1}$′, and $solu_{1}$′ is used to replace $solu_{1}$ in Figure 5c. As a result, the Maximum Transfer Time *MT* of the new recovery scheme in Figure 5c is reduced to 13 s.

**Time Complexity:** Initializing the recovery scheme involves selecting helper blocks and a storing rack for each stripe to be recovered. Specifically, for each stripe, it is necessary to select *k* helper blocks from *k* + *m* − 1 available data blocks and choose one rack from *r* racks for storing the recovery block. Therefore, the time complexity for initializing the recovery scheme for *s* stripes is $O\left(C\left(k+m-1,k\right)\cdot r\cdot s\right)$. In the worst case, optimizing the recovery scheme also requires traversing all recovery schemes for all stripes to be recovered. However, thanks to the Help array set up by HBRepair, it is unnecessary to enumerate all possible recovery schemes for each stripe. Instead, by utilizing the Help array, which records the status of each stripe’s data blocks saved on various racks, we can enumerate all recovery schemes that potentially minimize the maximum transmission time. Consequently, the time complexity for optimizing the multi-stripe recovery scheme can be reduced to $O(r^{2}\cdot s)$. In summary, the overall time complexity of Algorithm 1 is $O(C\left(k+m-1,k\right)\cdot r\cdot s+r^{2}\cdot s)$.

**Space Complexity:** The space complexity of Algorithm 1 consists of several components. Firstly, the Help array for recording the status of each stripe’s data blocks on different racks has a space complexity of O(r·s). Secondly, it is necessary to store the recovery schemes for *s* stripes to be restored, which include *k* helper blocks and one storage rack for each stripe, leading to a space complexity of O(s·(k+1)). Moreover, it is necessary to separately record the upload and download times for each rack, contributing to a space complexity of O(2·r). Lastly, the array for storing the transmission times between racks has a space complexity of $O(r^{2})$. In summary, the total space complexity of Algorithm 1 is $O(r\cdot s+s\cdot \left(k+1\right)+2\cdot r+r^{2})$.

### 3.3. Restore Abstraction

**Construction of HBDAG:** This paper first formalizes the recovery scheme for individual stripes in the form of a Directed Acyclic Graph (DAG), termed as the Recovery DAG for the HBrepair method (HBDAG). For RS(*k*,*m*), the HBDAG for a single stripe can be initialized with *k* + 1 vertices, where *k* vertices $\{v_{1},v_{2},\ldots ,v_{k}\}$ represent the nodes storing the helper blocks for recovery, and $v_{k+1}$ represents the node storing the recovery block. Additionally, this paper uses directed edges between vertices to represent the data transfer directions specified in the recovery algorithm.

This paper constructs these edges based on the following rules. Given two vertices $v_{i}$ and $v_{j}$(1≤i≠j≤k+1), a directed edge $e_{i,j}$ is used to represent the sending of a helper block from $v_{i}$ to $v_{j}$. Therefore, if $e_{i,j}$ exists, $v_{i}$ is referred to as the child node of $v_{j}$, and conversely, $v_{j}$ is the parent node of $v_{i}$. Vertex $v_{j}$(j≠k+1) can only send the result to its parent node after receiving all the required helper blocks and partial decoding them with the local data block, as shown in Equation (1). Since batch recovery involves recovering multiple stripes with damaged blocks, a node may simultaneously be the parent and child nodes of different HBDAGs.

**The recovery process guided by HBDAG:** The recovery commences from the leaf vertices, i.e., vertices without child nodes, and concludes at vertex $v_{k+1}$. For each edge $e_{i,j}$, once $v_{i}$ has transmitted the helper block to $v_{j}$ for recovery, the edge $e_{i,j}$ is removed from the HBDAG. For every leaf vertex, if no edges are connected to it, indicating that the leaf vertex has sent all helper blocks to its parent node, it should also be removed from the HBDAG. Thus, as the recovery proceeds, the vertices within the HBDAG diminish gradually until vertex $v_{k+1}$ becomes a leaf vertex. At this time, the recovery of the individual stripe corresponding to the HBDAG is accomplished.

**Figure 6:**
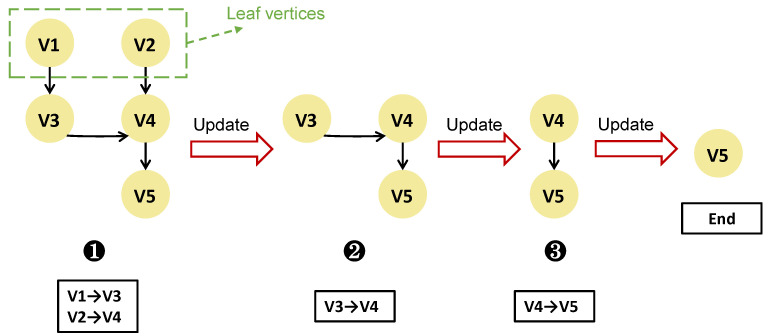
Example of HBDAG recovery using PPR method.

**Example analysis:** This paper takes the HBDAG recovered using PPR [4] method in Figure 6 as an example, where *k* = 4. Assume the data blocks stored in the four vertices $\{v_{1},v_{2},v_{3},v_{4}\}$ are represented by $\{C_{1},C_{2},C_{3},C_{4}\}$, respectively. In the first phase, vertex $v_{1}$ sends a data block $\beta_{1}C_{1}$ to its parent node $v_{3}$ (where $\beta_{1}$ is the decoding coefficient of $C_{1}$), and at the same time, $v_{2}$ sends another data block $\beta_{2}C_{2}$ to its parent node $v_{4}$. After completing the above data transfer, the HBDAG is updated by removing edge $e_{1,3}$, edge $e_{2,4}$, vertex $v_{1}$, and vertex $v_{2}$. In the second phase, the leaf vertex $v_{3}$ first combines the received $\beta_{1}C_{1}$ with its locally stored data block $C_{3}$, and then sends the result $\beta_{1}C_{1}$ + $\beta_{3}C_{3}$ to its parent node $v_{4}$. After the data transfer is completed, the HBDAG is updated by removing edge $e_{3,4}$ and vertex $v_{3}$. In the third phase, the leaf vertex $v_{4}$ combines the received result with its locally stored data block $C_{4}$ to recover the corrupted block $C^{\prime }=\sum_{i=1}^{4}\beta_{i}C_{i}$, and then sends C’ to $v_{5}$. Finally, after updating the HBDAG by removing edge $e_{4,5}$ and vertex $v_{4}$, $v_{5}$ becomes the leaf vertex, at which point the recovery of the corrupted block *C′* is completed.

**Advantages of HBDAG:** HBDAG is a general formalization of the single-strip recovery scheme, which means that once the recovery scheme and data transfer strategy are determined, an HBDAG can be constructed for any given recovery algorithm. Therefore, HBRepair is applicable to a variety of erasure codes (such as RS codes, LRC codes, and regenerating codes) and various single-strip recovery algorithms (such as CR, PPR [4], and ECPipe [22]). This design overcomes the limitation of being deployable only with specific recovery algorithms, achieving generality and flexibility in recovery.

### 3.4. Link Scheduling

In Algorithm 1, this paper reduces the Maximum Transfer Time *MT* by finding a near-optimal multi-strip recovery scheme. However, as described in Section 2.3, randomly scheduling links may result in a recovery time *CT* that is greater than the Maximum Transfer Time *MT*, making it crucial to arrange the execution order of recovery links reasonably. Based on this, the paper designs Algorithm 2, whose main idea is to first group the links that recover the same damaged block together, and then prioritize the scheduling of congested links within the groups where the strip recovery time is longer.
**Algorithm 2** Schedule repair links**Input:** number of stripes *s*, a list of cross-rack repair links *allLinks***Output:** the execution order of all recovery links in the cross-rack repair link list *allLinks* 1:   Compute the $Ru_{m}$ and $Rd_{m}$ for each rack $R_{m}$ 2:   Compute the $CL_{mn}$ for each link $R_{m}$ → $R_{n}$ in *allLinks* 3:   Divide the links that repair the same stripe $S_{i}$ into a group $Link_{i}$ 4:   **for** i=1 to *s* **do** 5:      Sort all links $R_{m}$ → $R_{n}$ in $Link_{i}$ in descending order based on $CL_{mn}$ 6:      Calculate the time $rTime_{i}$ for repairing a single strip $S_{i}$ 7:      Calculate the total upload link sum $Su_{i}$ for all the upload racks involved in the group $Link_{i}$ 8:   Sort each group $Link_{i}$ in descending order by $rTime_{i}$. (If there are groups with the sa-me repair time for a single strip, sort these groups in ascending order based on the total upload link sum of the upload racks involved within each group) 9:   send_rack=∅,recv_rack=∅,tran_link=∅   link_execute$\left[\mathrm{total}\;\mathrm{number}\;\mathrm{of}\;\mathrm{repair}\;\mathrm{links}\right]=\left\{0\right\}$10:   **while** *allLinks* ≠∅ **do**11:      **for** i=1 to *s* **do**12:         **for** each link $R_{m}$ → $R_{n}$ in $Link_{i}$ **do**13:            **if** $\mathrm{send}\_\mathrm{rack}\cap \{R_{i}\}=\emptyset$ **and** $\mathrm{recv}\_\mathrm{rack}\cap \{R_{j}\}=\emptyset$            **and** ${\mathrm{link}\_\mathrm{execute}}_{mn}=0$ **then**14:               startTransfer($R_{m}$ → $R_{n}$)15:               send_rack += $\left\{R_{m}\right\}$, recv_rack += $\left\{R_{n}\right\}$16:               $\mathrm{link}\_execute_{mn}$ = 117:               **break**18:      **for** each link $R_{m}$ → $R_{n}$ in tran_link **do**19:         **if** link $R_{m}$ → $R_{n}$ belong to $Link_{i}$ has completed transmission **then**20:            tran_link -= {$R_{m}$ → $R_{n}$}21:            send_rack -= $\left\{R_{m}\right\}$, recv_rack -= $\left\{R_{n}\right\}$22:            **if** there is a link in $Link_{i}$ can be transmitted **then**23:               Jump to the $Link_{i}$ scheduling of the for-loop in line 1124:            **else**25:               Jump to other groups links scheduling of the for-loop in line 10

This paper sets two counters, $Ru_{m}$ and $Rd_{m}$, for each rack $R_{m}$, which serve as the number of upstream and downstream visits to that rack, respectively. The congestion level of a link $R_{m}$ → $R_{n}$ is defined as $CL_{mn}$ = ($R_{m}$ + $R_{n}$) · $T_{mn}$, where $T_{mn}$ is the transmission time of the link $R_{m}$ → $R_{n}$. The congestion level of a link reflects the bandwidth usage situation; a higher congestion level indicates that the link occupies more bandwidth resources. The following will provide a detailed explanation using Table 4 and Table 5 as examples. In Table 4, there are 6 racks $\{R_{1},R_{2},R_{3},R_{4},R_{5},R_{6}\}$ and 13 recovery links $\left\{R_{1}\to R_{4}\left(5\;s\right),R_{2}\to R_{4}\left(4\;s\right),R_{3}\to R_{4}\left(3\;s\right),R_{3}\to R_{5}\left(3\;s\right),R_{4}\to R_{5}\left(4\;s\right),R_{6}\to R_{5}\left(5\;s\right),R_{3}\to R_{2}\left(2\;s\right),R_{4}\to R_{2}\left(3\;s\right),R_{5}\to R_{2}\left(4\;s\right),R_{2}\to R_{6}\left(3\;s\right),R_{3}\to R_{6}\left(3\;s\right),R_{4}\to R_{6}\left(3\;s\right),R_{5}\to R_{6}\left(3\;s\right)\right\}$, and the links highlighted in blue in the table are the recovery links involved in this example. As shown in Table 5, the upstream access counts ($Ru_{m}$) and downstream access counts ($Rd_{m}$) for the 6 racks $R_{m}$ are 1, 2, 4, 3, 2, 1 and 0, 3, 0, 3, 3, 4, respectively. Then, calculate the congestion level $CL_{mn}$ of the link $R_{m}$ → $R_{n}$. For example, the congestion level for the link $R_{1}$ → $R_{4}$, $CL_{14}$, is calculated as ($Ru_{1}$ + $Rd_{4}$) · $T_{14}$ = (1 + 3) · 5 = 20. Next, this paper will arrange the link scheduling order reasonably based on the congestion levels of each link.

**Algorithm details:** Before elaborating on the specific steps of Algorithm 2, it is necessary to explain that the number of lines in the following description refers to the number of lines of code in Algorithm 2. Given a set of recovery links *allLinks*, this paper first calculates the upstream and downstream access counts for each rack and the congestion level of each link (Lines 1–2 in Algorithm 2), and then groups the links according to the strips they are recovering (Line 3). The links within the same group are sorted in descending order based on their congestion level, and for each group, we calculate the single-strip recovery time, as well as the sum of the upload links for the upload racks within the group (Lines 4–7). The groups are then sorted in descending order based on their single-strip recovery time. If there are groups with the same single-strip recovery time, in order to make the total recovery time *BT* equal to the maximum transmission time *MT* as much as possible, the links in the groups with fewer selectable links per uploading rack on average should be prioritized for scheduling. This means these groups should be sorted in ascending order based on the sum of the upload links for the upload racks within the group (Line 8). Algorithm 2 sets up three variables and one array: *send_rack* and *recv_rack* record the racks currently sending and receiving data, respectively; *tran_link* records the link currently transmitting data; the *link_execute* array records whether each link has been scheduled. The three variables are initialized as empty sets, indicating that no racks are uploading or downloading data and no links are transmitting data; the elements in the *link_execute* array are initialized to 0, indicating that all links have not been scheduled yet (Line 9). Then, Algorithm 2 iterates through the links in each group in the order of the groups, and after sorting in descending order, the links with higher congestion levels in each group will be scheduled earlier (Lines 10–24). For the link $R_{m}$ → $R_{n}$, if both the uplink of $R_{m}$ and the downlink of $R_{n}$ are idle (not transmitting data) and the link $R_{m}$ → $R_{n}$ has not been scheduled, then the link can be initiated (Lines 11–14). At this point, $R_{m}$ is added to *send_rack*, $R_{n}$ is added to *recv_rack*, $R_{m}$ → $R_{n}$ is added to *tran_link*, and the corresponding element $\mathrm{link}\_execute_{mn}$ in the *link_execute* array for the link $R_{m}$ → $R_{n}$ is set to 1, indicating that the link has been scheduled (Lines 15–16). If the link in *tran_link* completes data transmission, the next link to be scheduled will be sought within the same group first. When there are no more links to be scheduled in that group, the next link to be scheduled will be sought in other groups according to the group order, and at the same time, the above three variables and the *link_execute* array will be updated (Lines 18–25). Algorithm 2 will repeat the above steps until all elements in the *link_execute* array are marked as 1, which means that all links have been scheduled and have completed data transmission (Line 10).

**Figure 7:**
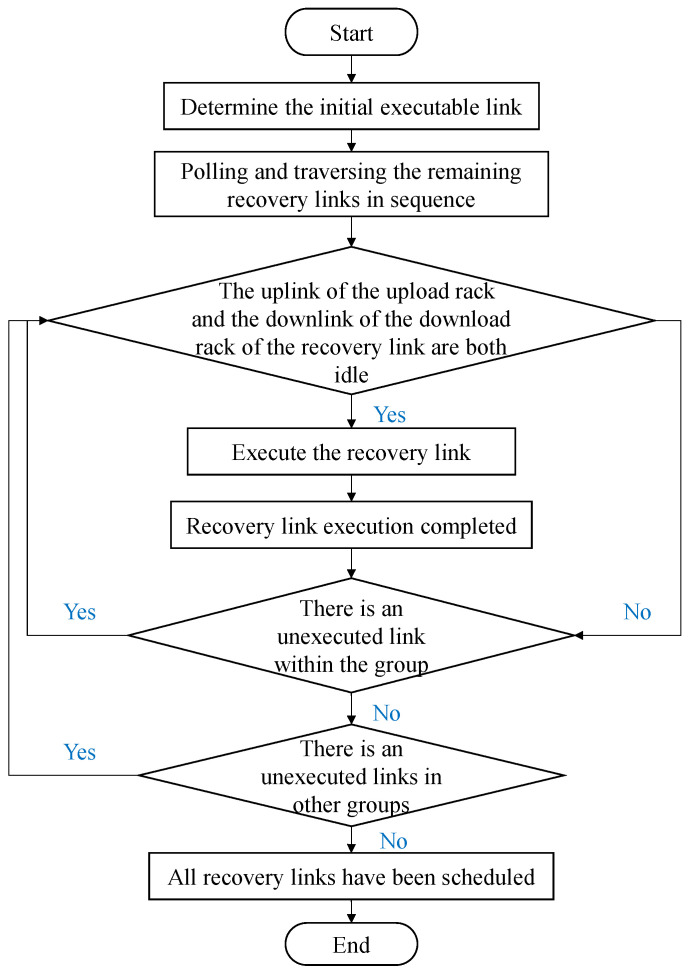
Partial flowchart of Algorithm 2.

**Algorithm flowchart:** Since the methods for calculating the number of uplink and downlink accesses for each rack, as well as for evaluating the congestion level of each recovery link in Algorithm 2, have been detailed in Table 4 and Table 5. At the same time, the grouping method of recovery links and the sorting logic for each group and the links within them have been thoroughly explained in the algorithm details. Therefore, as shown in Figure 7, this paper only provides the flow for the recovery link scheduling portion of Algorithm 2. The method for determining the initially executable recovery links follows the group order, sequentially selecting one executable recovery link from each group according to the link order. In this way, it can be ensured that at the beginning stage, each group has a recovery link in execution state.

**Figure 8:**
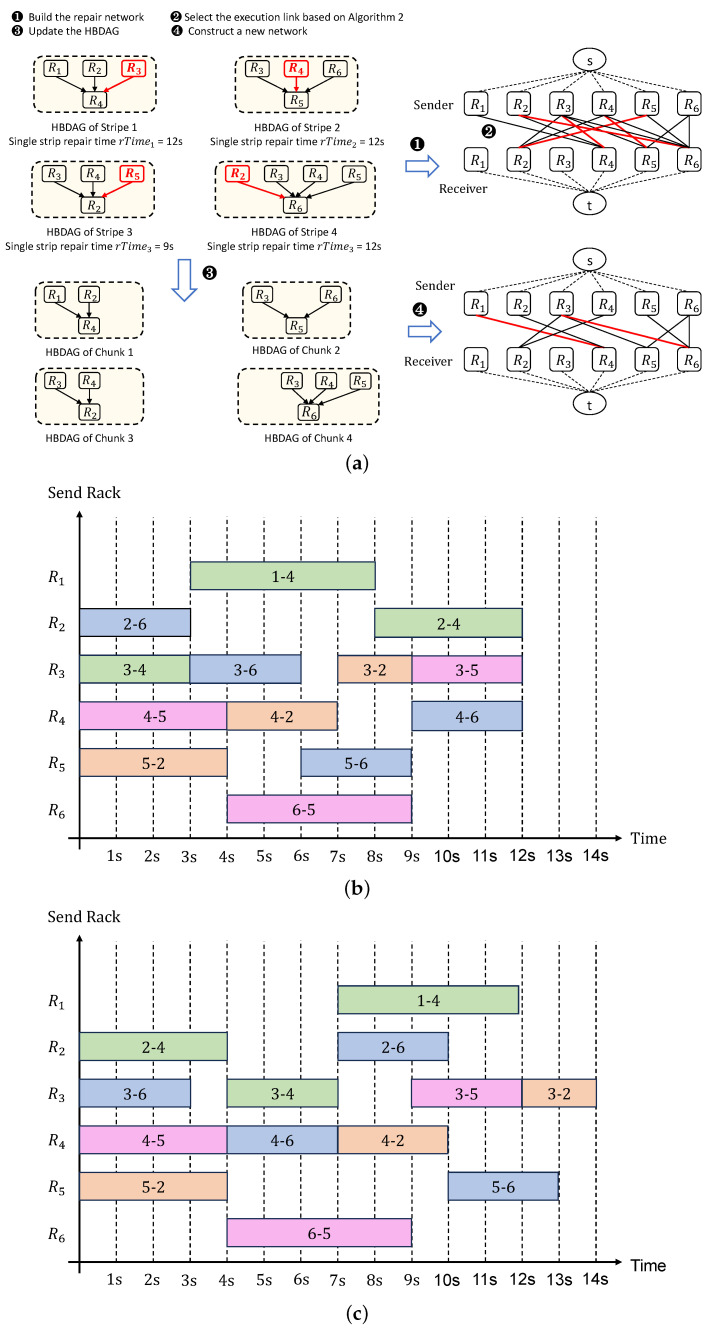
Analysis of Algorithm 2 application instance. (**a**) HBDAG for 4 stripes determined by Algorithm 1 using conventional recovery method; (**b**) Link scheduling order generated by HBRepair based on the HBDAGs in (**a**); (**c**) Link scheduling order generated by CMRepair based on the HBDAG in (**a**).

**Example analysis:** This paper provides an illustration of how Algorithm 2 operates through the examples presented in Table 4 and Table 5, Figure 8. Table 4 shows the cross-rack transmission times set for this example, which includes 6 racks and 13 cross-rack links (links marked in blue). Table 5 displays the calculation process for the link congestion level. Figure 8a shows the HBDAG determined by Algorithm 1 for four stripes with damaged blocks, with the red arrows in the figure indicating the scheduled links. It is evident that the HBDAG recovered using the CR method is presented here. Since cross-rack bandwidth is generally scarcer than cross-rack bandwidth, and according to the literature reviewed, the ratio of available cross-rack bandwidth to cross-rack bandwidth for each node is typically between 1/20 and 1/5, and in some extreme cases may drop to 1/240 [25,26,27], this paper assumes that the cross-rack transmission time is negligible, and it is necessary to convert the relationships between links and nodes in the HBDAG into relationships between links and racks before constructing the recovery network. This paper also assumes that when multiple helper blocks for recovering the same stripe are located in the same rack, these helper blocks need to be partially decoded locally before being sent to the rack where the recovery block is stored. Based on the above description, Figure 8a shows the HBDAGs established for 4 stripes with damaged blocks, and based on these 4 HBDAGs, a recovery network can be constructed on 14 vertices, which includes one source vertex *s*, one sink vertex *t*, 6 racks that may send helper blocks for recovery $\{R_{1},R_{2},R_{3},R_{4},R_{5},R_{6}\}$, and 6 racks that may store recovery blocks $\{R_{1},R_{2},R_{3},R_{4},R_{5},R_{6}\}$. Next, according to Algorithm 2, we begin to schedule the links. Since the recovery times for single stripes in Group 1, Group 2, and Group 4 are the maximum and equal, it is necessary to calculate the sum of the uplink access counts for the upload racks for each group separately: for Group 1, the sum of uplink access counts $Su_{1}$ is calculated as $Su_{1}$ = $Ru_{1}$ + $Ru_{2}$ + $Ru_{3}$ = 1 + 2 + 4 = 7; for Group 2, the sum of uplink access counts $Su_{2}$ is calculated as $Su_{2}$ = $Ru_{3}$ + $Ru_{4}$ + $Ru_{6}$ = 4 + 3 + 1 = 8; and for Group 4, the sum of uplink access counts $Su_{4}$ is calculated as $Su_{4}$ = $Ru_{2}$ + $Ru_{3}$ + $Ru_{4}$+ $Ru_{5}$ = 2 + 4 + 3 + 2 = 11. Because the sum of the uplink access times for the upload racks involved in Group 1 is the smallest, we prioritize scheduling the link with the highest congestion level in Group 1, which is $R_{3}$ → $R_{4}$. At this point, there are no schedulable links in Group 1, so we proceed to Group 2 to schedule the link with the highest congestion level within the group, which is $R_{4}$ → $R_{5}$, and so on. As a result, the first batch of executed links is {3–4, 4–5, 2–6, 5–2}. As shown in Figure 8b, where links within the same group are represented by the same color, the data transfer for both link $R_{2}$ → $R_{6}$ and link $R_{3}$ → $R_{4}$ is completed simultaneously. At this point, it is necessary to update the HBDAG for stripe 1 and stripe 4 first. Then, according to the group order, the unscheduled link with the highest congestion level and executable link $R_{1}$ → $R_{4}$ in the first group to which link $R_{3}$ → $R_{4}$ belongs is selected. Next, from the unscheduled links in the fourth group to which link $R_{2}$ → $R_{6}$ belongs, the link $R_{3}$ → $R_{6}$ with the highest congestion level and executable is selected, and so on. Based on Algorithm 2, the final link scheduling order obtained is {3–4, 4–5, 2–6, 5–2, 1–4, 3–6, 6–5, 4–2, 5–6, 3–2, 2–4, 3–5, 4–6}. As shown in Figure 8c, because CMRepair [6] does not group the links based on the stripes they recover, nor does it explicitly specify how to sort links with the same congestion level, the link scheduling order it obtains is {4–5, 3–6, 2–4, 5–2, 3–4, 4–6, 6–5, 1–4, 2–6, 4–2, 3–5, 5–6, 3–2}. This does not ensure that the links in the groups with the maximum single-stripe recovery time are executed without idleness, thus not minimizing the batch recovery time as much as possible. By comparison, it can be known that HBRepair reduces the batch recovery time from 14 s to 12 s compared to CMRepair [6], saving 14.3% of batch recovery time.

**Time complexity:** Firstly, calculate the number of uplink and downlink accesses for each rack, as well as the congestion level for each link. The time complexity for this part is O(2·k·s). Next, the recovered links are grouped into *s* groups based on the recovered stripes, with *k* links in each group. Then sort these groups in descending order of recovery time and sort the links within the group in descending order of congestion level. The time complexity of this part is O(s·log(s)+k·s·log(k·s)). Finally, traversing the recovery links by group using a round-robin method has a time complexity of O(k). In summary, the total time complexity of Algorithm 2 is O(2·k·s+s·log(s)+k·s·log(k·s)+k).

**Space Complexity:** The space complexity of Algorithm 2 consists of several components. First, it is necessary to record the number of uplink and downlink visits to each rack and the congestion level of each link, which has a space complexity of O(2·r+k·s). Second, it is required to keep track of whether each link has been scheduled, resulting in a space complexity of O(k·s). Additionally, the upload and download statuses of each rack need to be recorded, contributing a space complexity of O(2·r). In the best-case scenario for Algorithm 2, where both the upload and download links of each rack are transmitting data, up to *r* recovery links can be executed simultaneously; thus, the space complexity for recording the currently executing recovery links is O(r). In summary, the total space complexity of Algorithm 2 is O(5·r+2·k·s).

## 4. Expansion

In order to ensure the versatility and flexibility of the HBRepair recovery framework during the data recovery process, this paper extends it to be applicable to various erasure codes and recovery algorithms. The following section will elaborate on why HBRepair can be integrated with these different erasure codes and recovery algorithms.

**Applicable to various Erasure Codes:** As an orthogonal approach, HBRepair can be applied to different erasure codes, enabling rapid recovery in heterogeneous environment. In addition to RS codes, there are other commonly used erasure codes, such as LRC and MSR. Unlike RS codes, these erasure codes do not allow arbitrary selection of k helper blocks to recover a single failed block. For example, Butterfly code [28], as a type of minimum storage regenerating code, requires all the remaining *k + m* + 1 blocks to recover a single failed block. Although the determinism of the recovery scheme limits the performance of Algorithm 1 when applied to Butterfly code, as an orthogonal scheduling technique, Algorithm 2 can still provide a reasonable scheduling order for the determined recovery links, thereby reducing recovery time.

**Applicable to different recovery methods:** As a recovery framework, HBRepair is designed to apply to various recovery methods by implementing abstract recovery, such as CR (Conventional Recovery method), PPR [4], and ECPipe [22]. This flexibility allows HBRepair to select the most suitable recovery method for different deployment scenarios, thereby increasing the efficiency of batch recovery.

## 5. Performance Evaluation

This paper created nine cloud servers on Alibaba Cloud Elastic Compute Service (ECS), set up a Hadoop cluster, and conducted a multitude of experiments on Hadoop Distributed File System (HDFS) to compare and evaluate the performance of three recovery methods, namely, HBRepair, RepairBoost [5], and CMRepair [6].

### 5.1. System Architecture and Implementation

HBRepair consists of a global coordinator and multiple racks, where each rack contains one proxy and multiple nodes. The global coordinator is responsible for issuing recovery commands and managing the metadata of stripes, including the mapping relationship between data blocks and stripes, as well as the information of the nodes where each data block in the stripes is located. Additionally, the coordinator is in charge of managing the nodes that store the data blocks and the proxies within each rack. The proxies remain in a standby state, waiting for recovery commands from the coordinator and cooperating to execute the recovery operations.

This paper implements a prototype of HBRepair in C++, which consists of approximately 3500 lines of code. It functions as an independent middleware that operates on existing storage systems and can replace them to perform recovery operations when the storage system needs to be restored, thereby simplifying the complexity of storage systems and improving recovery efficiency. The paper realizes encoding and decoding functionalities based on the coding library Jerasure v2.0 [29] and transfers data via the Redis interface.

### 5.2. Experimental Design and Result Analysis

This paper created nine cloud servers ECS on Alibaba Cloud, set up a Hadoop 3.1.1 cluster, and evaluated HBRepair on HDFS. The instances are located in North China 2 (Beijing, China), each equipped with two Intel Xeon Platinum vCPUs at 2.5 GHz, 16 GB of memory, and 100 GB of ESSD cloud disk storage, running the CentOS 7.9 operating system. The following are the default configurations. The paper generates 60 stripes and uses RS (*k* = 6, *m* = 3) coding, which is used for Hadoop HDFS [2] and QFS [26], with each data block size set to 64 MB. Due to experimental limitations, only 9 nodes are available. In order to simulate a real rack architecture and reproduce cross-rack data transmission scenarios, ensuring that each rack contains at least 3 nodes, this paper evenly distributes these 9 nodes across 3 racks. The bandwidth within the rack is 1024 Mb/s, and then the Linux traffic control tool tc is used to limit the cross-rack bandwidth, thus creating bandwidth diversity in the data center [25,30]. Specifically, the paper varies the cross-rack bandwidth from 64 Mb/s to 512 Mb/s, making the ratio of cross-rack bandwidth to cross-rack bandwidth range between 2 and 16. In this environment, the paper compares HBRepair with two other recovery techniques, RepairBoost and CMRepair. This paper chooses these two recently proposed new methods as comparative objects because they have high representativeness and significant comparative value in batch recovery technology based on erasure codes. And after extensive research, this paper can confirm that they are currently the two best benchmark testing methods. Selecting them for comparison can fully validate the performance of HBRepair. Data blocks are randomly distributed across the entire rack, and one data block from each stripe is deleted to simulate a failure. All results are averaged over three runs, and recovery times are reported in seconds. The paper conducted five groups of experiments. First, the impact of parameter variations on HBRepair performance was analyzed in Experiment 1 and Experiment 2. Then, the flexibility, versatility, and effectiveness of HBRepair were demonstrated by deploying it on representative erasure codes and recovery algorithms in Experiment 3 and Experiment 4. Finally, the paper verified whether the two design techniques of HBRepair are complementary in Experiment 5.

**Figure 9:**
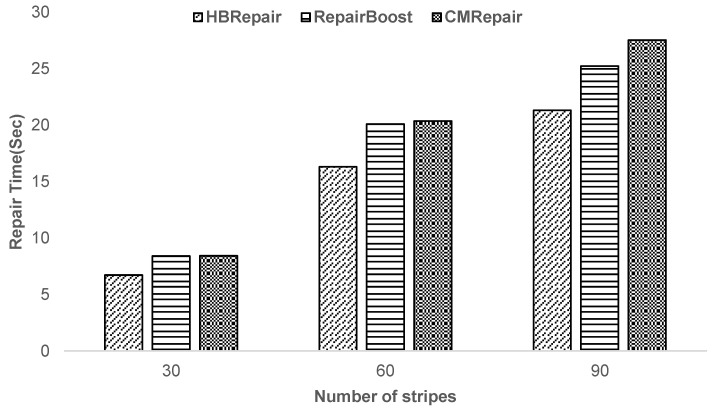
Impact of the number of stripes to be recovered on recovery time.

**Experiment 1—Analysis of the impact of the number of stripes to be recovered on HBRepair’s recovery performance:** As shown in Figure 9, with the increase in the number of stripes to be recovered, HBRepair’s recovery time significantly outperforms RepairBoost and CMRepair. CMRepair almost needs to traverse all recovery schemes for each stripe to be recovered when optimizing the recovery scheme. In contrast, HBRepair simplifies this process by introducing the “Help Array” and “Request Array” in Algorithm 1. HBRepair only needs to search for other recovery schemes consisting of help blocks from other racks except for the rack with the longest upload time for each strip to be recovered in the “Help array”. Or, in the “Request array”, HBRepair searches for other recovery schemes consisting of target nodes from racks other than the rack with the longest download time. Moreover, because helper blocks stored in the same rack have the same transfer time when sent to the target node, when a rack contains multiple helper blocks, only the recovery scheme consisting of one of the helper blocks for a single stripe needs to be considered. This greatly reduces the number of recovery schemes that each stripe to be recovered needs to traverse, decreasing the computation time of Algorithm 1, and this advantage becomes more pronounced as the number of stripes increases. RepairBoost only considers the recovery links connecting the leaf vertices (vertices without upload and download tasks) locally when scheduling the execution order of recovery links. In contrast, HBRepair takes a global approach, considering the scheduling of all recovery links, which allows it to perform recovery tasks more efficiently when dealing with a large number of stripes to be recovered. When recovering multiple stripes (30, 60, 90), HBRepair’s recovery time is reduced by 15.52–20.08% and 19.91–22.53% compared to RepairBoost and CMRepair, respectively. Upon analyzing Experiment 1, this paper observes that the improvement effect of HBRepair is relatively similar under different numbers of stripes, and the influence of stripe number on performance is not significant. Therefore, in designing the experiment, a compromise stripe number of 60 is selected as the default setting.

**Figure 10:**
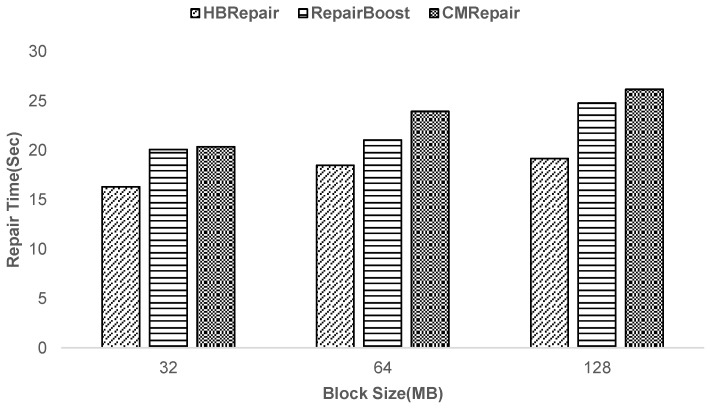
Impact of block size on recovery time.

**Experiment 2—Analysis of the impact of block size on HBRepair’s recovery performance:** To investigate the effect of block size on the recovery performance of HBRepair, this paper conducts experiments with different block sizes, namely, 32 MB, 64 MB, and 128 MB, and records the recovery times. Figure 10 shows the recovery times for different recovery techniques as the block size varies from 32 MB to 128 MB. The recovery time of HBRepair is reduced by 12.16–22.62% compared to RepairBoost and 19.91–26.74% compared to CMRepair. Observations indicate that as the block size increases, HBRepair’s recovery time decreases more significantly compared to that of RepairBoost [5] and CMRepair [6], primarily due to the multi-threaded pipelining processing technique and the flexible link scheduling strategy adopted by HBRepair during the recovery process. Firstly, HBRepair significantly improves recovery efficiency by parallel processing of disk I/O and data transfer through multi threading. Secondly, in terms of link scheduling, HBRepair prioritizes the scheduling of links within the same recovered stripe group, a method that is more efficient than RepairBoost’s approach of scheduling only leaf links (where the upload node only uploads data blocks without downloading data blocks) and CMRepair’s method of scheduling all recovery links uniformly. This also enables HBRepair to schedule recovery links more flexibly, maximizing the continuous execution of each recovery link without idle time and making full use of network bandwidth resources. Especially when the size of the data block increases, that is, when the execution time of the recovery link is prolonged, HBRepair’s scheduling strategy can highlight its advantages more effectively. It will not cause some links to be in a waiting state for execution for a long time due to improper scheduling, thus effectively shortening the recovery time.

**Figure 11:**
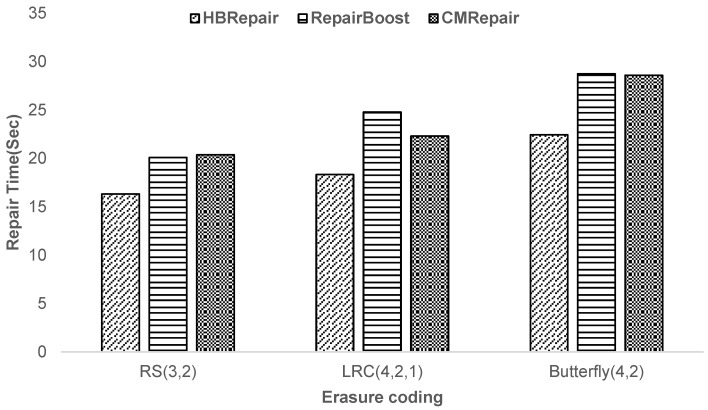
Analysis of HBRepair’s recovery performance across various Erasure Codes.

**Experiment 3—Analysis of HBRepair’s recovery performance across different erasure codes:** This paper uses representative erasure codes to verify the versatility of HBRepair. (i) Traditional RS(*k*,*m*), the most classic and popular erasure code. (ii) LRC(*k*,*l*,*g*), which divides *k* blocks into *l* groups. (iii) Butterfly(*k*,2), the erasure code with the minimum recovery traffic. As shown in Figure 11, compared to RepairBoost and CMRepair, HBRepair can reduce recovery time by an average of 22.25% and 19.74% for different erasure codes. Under the same encoding parameters, when HBRepair is applied to the Butterfly code, which requires all *k + m* + 1 remaining blocks to complete recovery, the determinism of the recovery scheme results in Algorithm 1 (helper block selection) having a relatively weaker impact on recovery performance. In contrast, the importance of Algorithm 2 (scheduling recovery links) becomes more pronounced. As Figure 11 indicates, HBRepair still maintains the performance improvement brought by Algorithm 2. This is because the second design technique not only ensures the parallel efficiency of data transmission but also optimizes the use of upload and download bandwidth, bringing it to saturation and effectively avoiding network congestion issues. Thus, it is proven that HBRepair can be applied to different erasure codes to facilitate recovery.

**Figure 12:**
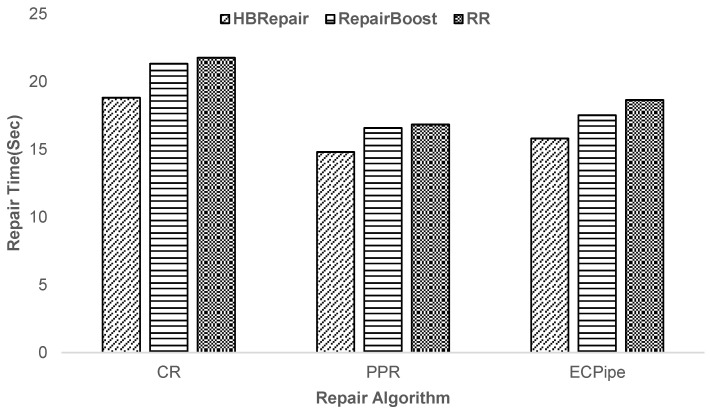
Analysis of HBRepair’s recovery performance across various recovery algorithms.

**Experiment 4—Analysis of HBRepair’s recovery performance with different recovery algorithms:** Since CMRepair cannot be applied to different recovery algorithms, in this part of the experiment, the paper replaces the comparison object with the recovery methods for randomly generating recovery schemes and scheduling recovery links randomly, abbreviated as RR. As shown in Figure 12, for RS(3,2), the PPR recovery algorithm has the least recovery time, while the conventional CR recovery algorithm has the most recovery time. The primary reason is that the PPR [4] algorithm enables parallel transmission of data blocks, while the ECPipe [22] algorithm also enhances recovery efficiency through data block slicing and pipelined recovery techniques. In contrast, the conventional CR recovery algorithm, which can only transmit one data block at a time, severely hampers its parallel processing capability, thereby making PPR and ECPipe significantly superior to CR in terms of recovery efficiency. Overall, compared to RepairBoost and RR, HBRepair can reduce the recovery time by an average of 10.77% and 14.17% for different recovery algorithms.

**Figure 13:**
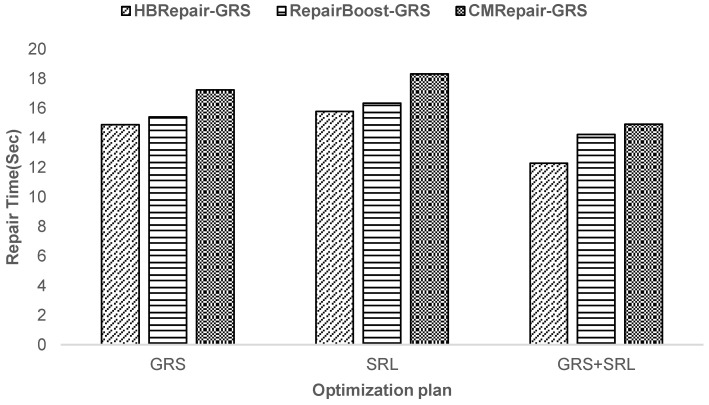
Decomposition analysis of two algorithm designs for HBRepair.

**Experiment 5—Analysis of recovery performance under the individual effects of two design techniques:** This paper conducts a decomposition analysis of HBRepair to demonstrate the effectiveness of each design technique. For simplicity, each technique of HBRepair is abbreviated as follows. (i) Generating Recovery Scheme (GRS), which produces a near-optimal recovery scheme to reduce the maximum transfer time (Algorithm 1, Section 3.2). (ii) Scheduling Recovery Links (SRL), which arranges the execution order of recovery links in a reasonable manner (Algorithm 2, Section 3.4). (iii) GRS+SRL, which is HBRepair. When using the GRS technique alone, the recovery links are scheduled randomly. When using the SRL technique alone, only the initialization process of Algorithm 1 is executed simply. Figure 13 shows the comparative results. First, it can be observed that the performance of SRL is superior to that of GRS, indicating that optimizing the scheduling order of recovery links is more effective than optimizing the recovery scheme. Second, HBRepair has the shortest recovery time, which is 12.87% and 17.9% less than GRS and SRL, respectively. This result indicates that these two optimization techniques complement each other, do not offset each other’s effectiveness, and instead enhance the overall recovery performance.

### 5.3. Theoretical Analysis

Due to the limitations of experimental conditions, this paper is unable to evaluate HBRepair in a real large-scale cluster. Therefore, in this section, this paper will evaluate the effectiveness of HBRepair in a larger-scale environment, as well as its performance in the worst-case scenario, through theoretical analysis. This section will also theoretically quantify the computational cost of HBRepair by combining the time complexity analysis of the HBRepair algorithm detailed in Section 3 and compare it with CMRepair and RepairBoost to demonstrate that the trade-off between cost and performance of HBRepair is reasonable.

**The effectiveness evaluation of HBRepair:** As analyzed in Section 3, the total time complexity of the HBRepair algorithm is $O(C\left(k+m-1,k\right)\cdot r\cdot s+r^{2}\cdot s)$ + O(2·k·s+s·log(s)+k·s·log(k·s)+k). Although it appears complex, a deeper analysis reveals that even in the worst-case scenario, where the scale of the distributed storage system is extremely large and both the number of racks *r* and the total number of recovery stripes *s* are exceptionally large, the performance of the HBRepair algorithm can still be guaranteed.

First, the combination number *C*(*k* + *m* − 1, *k*) is an important factor affecting the algorithm’s complexity. As *k* and *m* increase, the value of *C*(*k* + *m* − 1, *k*) grows rapidly. However, in practical applications, the coding parameters *k* and *m* of erasure codes are typically set based on specific scenarios and the importance of data and are not very large, keeping the growth of *C*(*k* + *m* − 1, *k*) within a controllable range.

Second, the product term of the number of racks *r* and the total number of recovery stripes *s* occupies a significant position in the algorithm’s complexity, as these two parameters directly reflect the scale of the system. Although in the worst-case scenario, the increase in *r* and *s* will significantly raise the algorithm’s complexity, thanks to the data parallel processing characteristics of distributed systems, the recovery tasks are effectively distributed, thus maintaining the overall system performance and not being severely limited by the increased complexity.

As for the lower-order terms such as k·s and s·log(s), their growth rates are relatively slow, and their impact on the overall complexity is minor. Therefore, in a large-scale distributed storage system with reasonable configuration, the performance of the HBRepair algorithm can be fully guaranteed, without leading to unacceptable time overhead.

**Computational Cost analysis of HBRepair:** In generating the recovery scheme, this paper introduces HBDAG and an optimized recovery scheme component to minimize the maximum transmission time of the recovery scheme. Due to the relatively low time complexity of creating HBDAG, this paper ignores its impact on the total computational cost. The main computational cost of HBRepair lies in initializing the recovery scheme and optimizing the recovery scheme, with a total time complexity of $O\left(C\left(k+m-1,k\right)\cdot r\cdot s\right)+O\left(r^{2}\cdot s\right)$. To validate the reasonableness of this additional computational overhead, this paper compares the computational cost of HBRepair with two baseline methods—CMRepair and RepairBoost.

By analyzing the algorithm of CMRepair, it can be seen that its computational cost for generating the recovery scheme is $O\left(2\cdot C(k+m-1,k)\cdot r\cdot s\right)$, which theoretically is significantly higher than that of HBRepair, indicating that HBRepair has certain advantages in computational cost over CMRepair.

In the original paper of RepairBoost, the total time complexity for generating the recovery scheme is found to be $O\left(s\cdot \left(|T|\cdot \log(|T|)+n\cdot |T|\cdot \log(n)\right)\right)$, where |T| represents the number of nodes involved in intermediate calculations, and *n* is the total number of nodes involved in recovery. For ease of comparison, this paper scales the number of nodes involved in intermediate calculations to the total number of nodes in the cluster (as the two are of similar orders of magnitude, this conversion is within a reasonable range), resulting in a converted complexity of $O\left(s\cdot n\cdot \log(n)\cdot (1+n)\right)$. Although in cluster architectures, the number of racks *r* is usually less than the number of nodes *n*, in the worst-case scenario where *r* is close to *n*, the computational cost of HBRepair might be slightly higher than that of RepairBoost, but this difference is relatively small. More importantly, by analyzing the results of Experiment 1, it can be understood that the advantage of HBRepair in recovery performance is not reflected in the generation of the recovery scheme but is more evident in the optimization of the link scheduling strategy. Therefore, even in the worst-case scenario where the computational cost of HBRepair is comparable to that of RepairBoost, its recovery performance still surpasses the latter.

In conclusion, the HBRepair algorithm achieves a reasonable and effective trade-off between computational cost and recovery performance. By introducing a relatively small computational overhead, it enhances recovery performance, providing an efficient and practical solution for the field of cluster failure recovery.

## 6. Conclusions

This paper considers cross-rack batch recovery techniques in heterogeneous erasure coding clusters and proposes an efficient cross-rack batch recovery framework named HBRepair, which can be applied to various erasure codes and recovery algorithms. The main idea is to formalize single-block recovery schemes using a directed acyclic graph called HBDAG. Then, recovery tasks are carefully allocated to specific nodes based on HBDAG to balance upload and download recovery traffic. HBRepair also arranges the execution order of recovery links reasonably to saturate unoccupied upload and download bandwidth. Through large-scale experiments on the Alibaba Cloud platform, this paper verifies the application of HBRepair in RS code, LRC code, MSR code, as well as CR algorithm, PPR algorithm, and ECPipe algorithm, confirming its generality, flexibility, and effectiveness. Compared to RepairBoost and CMRepair, HBRepair can reduce recovery time by 11.64–26.74%. In future work, HBRepair can be extended to utilize an SDN controller to monitor and obtain real-time dynamic status information of the network, and adjust recovery schemes in real time based on this information, thereby enhancing the recovery efficiency in response to dynamic events such as network load fluctuations.

In future work, this paper also plans to conduct experiments in a larger-scale cluster environment, increasing the number of nodes to simulate a more realistic large-scale distributed storage system, thereby providing a more comprehensive and rigorous validation of the algorithm. We will also design corresponding solutions to deal with possible abnormal situations during the recovery process, such as network failures and node failures, to ensure that the recovery operation can proceed continuously and without interruption.

## Figures and Tables

**Table 1:** Glossary.

Terminology	Parameters	Definition and Explanation
RS(k,m)	*k*: Number of data blocks *m*: Number of parity blocks	Reed-Solomon (RS) code is a type of erasure code that can tolerate the loss of up to *m* blocks and recover the original data from the remaining *k* data blocks.
LRC(k,g,l)	*k*: Number of global data blocks *g*: Number of global parity blocks *l*: Number of local parity blocks	LRC (Locally Repayable Codes) code is also a type of erasure code that adds additional checksum information within a local range, allowing each lost or damaged data block to be recovered by accessing the local checksum block instead of the entire checksum block during data recovery.
MSR(*n*,*k*,*d*,α,β)	*n*: Total number of nodes *k*: Minimum number of nodes required for reconstruction *d*: Number of nodes connected for repair α: Amount of data stored per node β: Amount of data transmitted by helper nodes	MSR (Minimum Storage Regenerating) is an optimized RS code that is particularly suitable for distributed storage systems. It reduces recovery latency and bandwidth consumption by minimizing the number of nodes that need to be accessed during data recovery.
Butterfly(*k*,2)	*k*: Number of data blocks	The Butterfly code is a type of MSR code that optimizes data placement and recovery processes based on a graph structure. This architecture permits efficient data recovery by accessing a minimal number of other blocks when a data block is lost or corrupted.

**Table 2:** Cross-rack transmission time during data recovery using the CR method.

Cross Rack Time	$R_{1}$	$R_{2}$	$R_{3}$	$R_{4}$	$R_{5}$
$R_{1}$	/	4	3	3	4
$R_{2}$	4	/	3	5	4
$R_{3}$	3	3	/	2	3
$R_{4}$	3	5	2	/	5
$R_{5}$	4	4	3	5	/

**Table 3:** Cross-rack data transfer time in the example using the PPR recovery method.

	Down	$R_{1}$	$R_{2}$	$R_{3}$	$R_{4}$	$R_{5}$	$R_{6}$
Up							
$R_{1}$		/	6 s	7 s	5 s	8 s	5 s
$R_{2}$		6 s	/	2 s	4 s	4 s	3 s
$R_{3}$		7 s	2 s	/	3 s	3 s	3 s
$R_{4}$		5 s	4 s	3 s	/	4 s	3 s
$R_{5}$		8 s	4 s	3 s	4 s	/	5 s
$R_{6}$		5 s	3 s	3 s	3 s	5 s	/

**Table 4:** Data transfer time between racks and recovery instances using standard recovery methods.

	Down	$R_{1}$	$R_{2}$	$R_{3}$	$R_{4}$	$R_{5}$	$R_{6}$
Up							
$R_{1}$		/	4 s	3 s	5 s	3 s	4 s
$R_{2}$		2 s	/	5 s	4 s	2 s	3 s
$R_{3}$		4 s	2 s	/	3 s	3 s	3 s
$R_{4}$		3 s	3 s	2 s	/	4 s	3 s
$R_{5}$		5 s	4 s	5 s	4 s	/	3 s
$R_{6}$		3 s	3 s	3 s	6 s	5 s	/

**Table 5:** Calculation process of congestion levels for each recovery link in the example.

$Ru_{1}$	$Ru_{2}$	$Ru_{3}$	$Ru_{4}$	$Ru_{5}$	$Ru_{6}$	
1	2	4	3	2	1	
$Rd_{1}$	$Rd_{2}$	$Rd_{3}$	$Rd_{4}$	$Rd_{5}$	$Rd_{6}$	
0	3	0	3	3	4	
$CL_{1,4}$	$CL_{2,4}$	$CL_{3,4}$	$CL_{3,5}$	$CL_{4,5}$	$CL_{6,5}$	
20	20	21	21	24	20	
$CL_{3,2}$	$CL_{4,2}$	$CL_{5,2}$	$CL_{2,6}$	$CL_{3,6}$	$CL_{4,6}$	$CL_{5,6}$
14	18	20	18	24	21	18

## Data Availability

Data are contained within the article.
